# Dopaminergic mesolimbic structural reserve is positively linked to better outcome after severe stroke

**DOI:** 10.1093/braincomms/fcae122

**Published:** 2024-04-09

**Authors:** Liv Asmussen, Benedikt M Frey, Lukas K Frontzkowski, Paweł P Wróbel, L Sophie Grigutsch, Chi-un Choe, Marlene Bönstrup, Bastian Cheng, Götz Thomalla, Fanny Quandt, Christian Gerloff, Robert Schulz

**Affiliations:** University Medical Center Hamburg-Eppendorf, Department of Neurology, 20246 Hamburg, Germany; University Medical Center Hamburg-Eppendorf, Department of Neurology, 20246 Hamburg, Germany; University Medical Center Hamburg-Eppendorf, Department of Neurology, 20246 Hamburg, Germany; University Medical Center Hamburg-Eppendorf, Department of Neurology, 20246 Hamburg, Germany; University Medical Center Hamburg-Eppendorf, Department of Neurology, 20246 Hamburg, Germany; University Medical Center Hamburg-Eppendorf, Department of Neurology, 20246 Hamburg, Germany; University Medical Center Hamburg-Eppendorf, Department of Neurology, 20246 Hamburg, Germany; University Medical Center Leipzig, Department of Neurology, 04103 Leipzig, Germany; University Medical Center Hamburg-Eppendorf, Department of Neurology, 20246 Hamburg, Germany; University Medical Center Hamburg-Eppendorf, Department of Neurology, 20246 Hamburg, Germany; University Medical Center Hamburg-Eppendorf, Department of Neurology, 20246 Hamburg, Germany; University Medical Center Hamburg-Eppendorf, Department of Neurology, 20246 Hamburg, Germany; University Medical Center Hamburg-Eppendorf, Department of Neurology, 20246 Hamburg, Germany

**Keywords:** brain reserve, voxel-based morphometry, cognition, grey matter, dopamine

## Abstract

The concept of brain reserve capacity has emerged in stroke recovery research in recent years. Imaging-based biomarkers of brain health have helped to better understand outcome variability in clinical cohorts. Still, outcome inferences are far from being satisfactory, particularly in patients with severe initial deficits. Neurorehabilitation after stroke is a complex process, comprising adaption and learning processes, which, on their part, are critically influenced by motivational and reward-related cognitive processes. Amongst others, dopaminergic neurotransmission is a key contributor to these mechanisms. The question arises, whether the amount of structural reserve capacity in the dopaminergic system might inform about outcome variability after severe stroke. For this purpose, this study analysed imaging and clinical data of 42 severely impaired acute stroke patients. Brain volumetry was performed within the first 2 weeks after the event using the Computational Anatomy Toolbox CAT12, grey matter volume estimates were collected for seven key areas of the human dopaminergic system along the mesocortical, mesolimbic and nigrostriatal pathways. Ordinal logistic regression models related regional volumes to the functional outcome, operationalized by the modified Rankin Scale, obtained 3–6 months after stroke. Models were adjusted for age, lesion volume and initial impairment. The main finding was that larger volumes of the amygdala and the nucleus accumbens at baseline were positively associated with a more favourable outcome. These data suggest a link between the structural state of mesolimbic key areas contributing to motor learning, motivational and reward-related brain networks and potentially the success of neurorehabilitation. They might also provide novel evidence to reconsider dopaminergic interventions particularly in severely impaired stroke patients to enhance recovery after stroke.

## Teaser

Asmussen *et al*. report that larger volumes of the dopaminergic key areas amygdala and nucleus accumbens are associated with a more favourable outcome after severe ischaemic stroke. This suggests that structural reserve of the mesolimbic system might play a role in recovery processes after severe stroke.

## Graphical Abstract

**Graphical Abstract:**
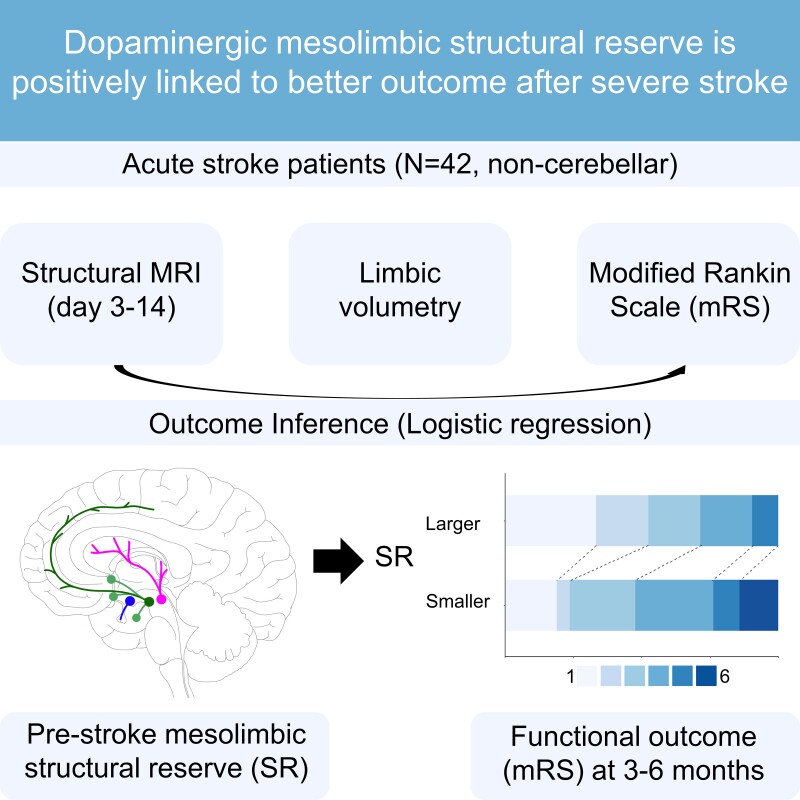

## Introduction

Stroke is one of the leading causes of persistent functional impairment worldwide.^1^ The outcome after stroke crucially affects patients’ quality of life and plays an increasing role in the public health of aging societies.^2^ Despite a substantial improvement in acute stroke treatment, a relevant proportion of stroke survivors is left with severe deficits. The amount of functional improvement over time varies between individual patients, the translation of current recovery models, obtained in cohorts with rather mild to moderate deficits, to patients with larger symptom burden remains unsatisfactory.^3,4^ There is still a relevant gap of knowledge about why some patients with severe deficits recover more than others, despite comparable clinical or demographic properties. Previous studies have aimed at exploring potential imaging^5^ or electrophysiological biomarkers to better understand outcome variability after a severe stroke. For instance, they have focused on lesion topography,^6^ early brain network alterations^7-9^ or motor cortical excitability^10^ to improve outcome models.

Aside from such parameters which relate more directly to the acute brain lesion itself, more recently, the field has moved towards the concepts of brain reserve capacity as a novel, informative measure for correlative or predictive outcome inference.^11-13^ According to this concept, individual structural and functional properties of the brain modulate the degree of impairment caused by a brain lesion by influencing the actual clinical manifestation.^14^ For instance, it might explain why younger stroke patients usually show a remarkably larger potential for recovery despite large and strategically located lesions. Global radiomics-derived surrogates of the pre-stroke, i.e. the pre-morbid state of the brain, have been successfully linked to outcome variability. Examples are relative brain age^15-17^ or more directly accessible estimates of brain health, such as white matter hyperintensity burden or the degree of brain atrophy.^18-20^ Besides these global parameters, other studies have focused on measures of focal structural brain reserve of distinct regions, such as the cerebellum^21^ or the cortex of the contralesional hemisphere.^22^ These analyses of brain reserve capacity have significantly added novel mechanistic insights into how pre-stroke anatomy of specific brain regions or networks might influence recovery processes.

**Figure 1:**
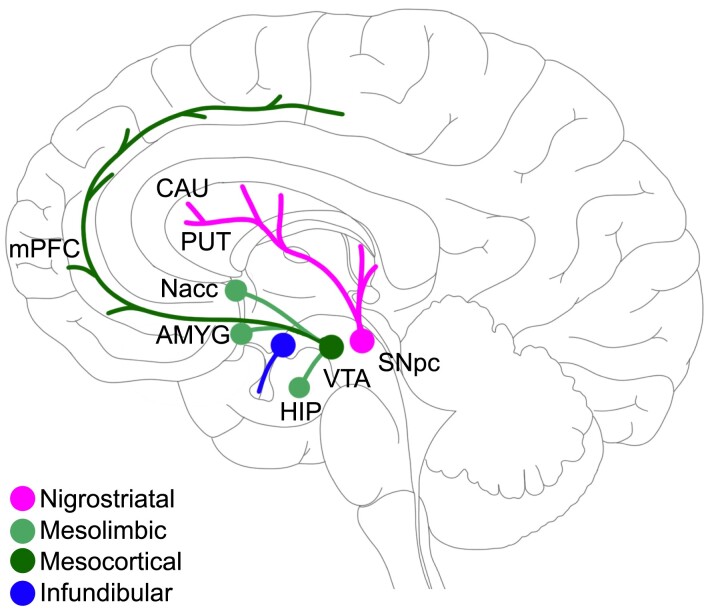
**Dopaminergic pathways.** This illustration gives an overview of the main dopaminergic pathways. Dopaminergic neurotransmission derives mainly from the pars compacta of the substantia nigra and the ventral tegmental area, but also from hypothalamus. Neurones from the substantia nigra project to the dorsal striatum including the putamen and the caudate nucleus whereas neurotransmission from the ventral tegmental area reaches limbic structures, such as the amygdala, the hippocampus, the nucleus accumbens and the media preofrontal cortex.

The rehabilitation after stroke is a complex process. Aside from functional adaptation, the re-learning of lost functions and the acquisition of novel skills are critically involved.^23,24^ On their part, these mechanisms are influenced by motivational and reward-related cognitive processes.^25,26^ In a recent randomized controlled trial, feedback and monetary reward in virtual-reality arm training positively influenced the improvement in motor scores in subacute stroke patients.^27^ One important neuronal system contributing not only to the motor domain, such as planning and execution of voluntary movements but also to the cognitive domain, including learning, motivation, reward and memory processes, is the dopaminergic system.^26,28-32^ It comprises of four major pathways (Fig. 1): the nigrostriatal, the mesolimbic, the mesocortical and the tuberoinfundibular pathway.^33^ The nigrostriatal pathway consists of dopaminergic neurones in the substantia nigra which project to the caudate nucleus (CAU) and putamen (PUT). Alterations in this pathway are not only fundamentally involved in Parkinson’s disease^34^ but also in motor planning, motor control and habit formation in healthy participants.^35,36^ The ventral tegmental area (VTA) projects via the mesolimbic pathways primarily to the amygdala (AMYG), the nucleus accumbens (Nacc) and the hippocampus (HIP),^37^ and via the mesocortical pathway to multiple cortical areas, predominantly the medial prefrontal cortex (mPFC).^31,38-40^ The mesolimbic and the mesocortical pathway together are commonly referred to as the mesocorticolimbic pathway.^31^ Finally, the tuberoinfundibular pathway comprises the dopaminergic innervation of the hypothalamic arcuate nucleus.^31,41^ Collectively, multiple deep subcortical nuclei, limbic brain regions and the medial prefrontal cortex are structural key areas of the dopaminergic system. They have been linked to motor and cognitive functions including motivational and reward processes which could exert an importance for neurorehabilitation.

The question arises whether the amount of structural reserve capacity in the dopaminergic system might inform about outcome variability after a severe stroke, i.e. in patients with the highest needs to regain lost neurological functions. For this purpose, this study analysed imaging and clinical data of 42 severely impaired acute stroke patients. Imaging data were collected within the first two weeks after stroke. Brain volumetry was performed to estimate grey matter volumes for seven key areas of the human dopaminergic system along the mesocortical, mesolimbic and nigrostriatal pathways of the contralesional hemisphere. Ordinal logistic regression models were used to relate regional volumes to the future functional outcome after 3–6 months after stroke, operationalized by the modified Rankin Scale (mRS). Models were adjusted for age, lesion volume and the initial impairment. We hypothesized that larger volumes of dopaminergic key areas are positively related to a more favourable outcome after a severe stroke.

## Materials and methods

### Participant data

**Figure 2:**
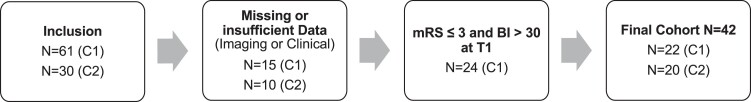
**Inclusion criteria and study integration.** The flowchart shows the integration process. Of the 91 patients that were considered in the beginning, 49 had to be excluded from the present analyses due to missing or insufficient data.

Clinical and imaging data taken from two independent cohorts of acute ischaemic stroke patients were re-analysed.^7,42^ Sixty-one patients were recruited for the first cohort (C1),^42^ and 30 patients for the second cohort (C2).^7^ All 91 patients were admitted to, and treated at the University Medical Center Hamburg-Eppendorf between 2012 and 2020. For proper integration of the two cohorts, inclusion criteria of both studies were combined. The final sample comprised of 42 more severely impaired stroke patients (C1: 22 patients, C2: 20 patients). A flow chart of cohort integration is given in Fig. 2. Inclusion criteria for both studies were age ≥ 18 years, first-ever unilateral ischaemic stroke, persistent motor deficit of the upper extremity including hand function and no history of severe psychiatric or neurological disease. Patients of cohort C1 were only included if Barthel Index was ≤30 or mRS > 3 at the acute stage (days 3–5). This approach of cohort integration was successfully applied by our previous studies.^21,22^ All patients underwent structural (T1-weighted) brain imaging (MRI) within the first two weeks after stroke (C1: days 3–5; C2: days 3–14). Baseline symptom burden was assessed using the National Institutes of Health Stroke Scale (NIHSS) at study inclusion (T1). In cohort C1, the follow-up timepoint (T2) was set after three months in the late subacute stage of recovery.^43^ Follow-up time in cohort C2 was set accordingly, but as clinical follow-up data for seven patients were only available six months after the event, T2 for these patients was set at six months post-stroke.^7^ Functional outcome at T2 was operationalized by the mRS, a measure of persistent global disability, in line with our previous reports.^21,22^

To compare brain volumetric data, 42 healthy controls of similar age and gender were included in this analysis. A pseudo-affected hemisphere was assigned to each control accordingly to account for the distribution of the lesion across right and left hemispheres. Informed consent was provided by all participants or their legal guardian in line with the ethical declaration of Helsinki. Original studies were granted permission by the local ethics committee of the Chamber of Physicians Hamburg (PV3777, PV5442 and PV5357).

### Brain imaging and voxel-based morphometry

All participants underwent brain imaging at the same 3T Skyra MRI scanner (Siemens, Erlangen, Germany). A 32-channel head coil and a magnetization-prepared rapid gradient echo sequence was used [parameters: time repetition (TR) = 2500 ms, time echo (TE) = 2.12 ms, flip angle = 9°, 256 coronal slices with a voxel size of 0.8 × 0.8 × 0.9 mm^3^, and field of view (FOV) = 240 mm]. T2-weighted images were performed to help delineate stroke lesions by using a fluid-attenuated inversion recovery sequence applying TR = 9000 ms, TE = 86 ms, time to inversion = 2500 ms, flip angle = 150°, 43 transversal slices with a voxel size of 0.7 × 0.7 × 3.0 mm^3^, and FOV = 230 mm as parameters. The Computational Anatomy Toolbox (CAT12)^44^ was used for voxel-based morphometry of the imaging data. The pipeline includes the steps of normalization, segmentation, data-quality-estimation and smoothing. First, the data were segmented according to the CAT12 manual. Individual images were displayed in SPM12 to check for possible gross anatomical abnormalities. To improve spatial registration, the images were centred manually at the anterior commissure and oriented to the anterior–posterior commissure line. In the next step, the images were segmented into white matter, grey matter and cerebrospinal fluid. The DARTEL algorithm was used for spatial normalization and modulation of the segmented images.^44,45^ Next, the images were transformed to the Montreal Neurological Institute (MNI) standard space. Finally, the normalized, modulated grey matter segmentations were smoothed with an 8-mm full-width at half-maximum Gaussian kernel to improve the signal-to-noise ratio. The total intracranial volume (TIV) was estimated by summing the volumes of grey matter, white matter and cerebrospinal fluid. Grey matter volumes of the following seven key structures of the dopaminergic system along the mesocorticolimbic and the nigrostriatal pathways were estimated based on AAL3 atlas^46^ data included in CAT12: nucleus accumbens, medial prefrontal cortex, amygdala, hippocampus, substantia nigra (pars compacta) (SNpc), caudate nucleus and putamen. For the medial prefrontal cortex, the label of the medial frontal gyrus was used. Data were obtained from the unaffected, contralesional hemisphere only to exclude any direct lesion effects.

### Statistical analysis

R version 4.2.1 was used to conduct the statistical analyses (r-project.org). Gender and handedness were compared between stroke patients and healthy controls using χ^2^ tests, mean age and size of the regional volumes were compared via unpaired two-sided *t*-tests. For volumetry-outcome inference in the stroke patients, we used ordinal logistic regression models fitted via ‘polr’ from the MASS package.^47^ The dependent variable was mRS at timepoint T2. The independent variables of interest were the regional volumes at timepoint T1 (one separate model for each region), dichotomized by median split into larger and smaller regional volumes, in line with our previous reports to improve statistical power and reduce the risk of influential points.^21,22^ Age, TIV, stroke lesion volume and NIHSS at timepoint T1 were added as covariates. Age and TIV were included after linear residualization against the volumes of interest to address multi-collinearities. Lesion volumes were log_10_-transformed to improve data distribution. Odds ratios (ORs) for the variables of interests were calculated along with the confidence intervals (CI, 95%) and the *P*-values. An OR < 1 would correspond to a lower risk for scoring one level higher in mRS in patients with larger volumes in comparison to patients with smaller volumes. All tests were conducted with a two-sided alpha level of 5% and corrected for multiple comparisons using the method of false discovery rate (noted as *P*_FDR_). A leave-one-out model analysis (LOOA) was finally applied to validate the robustness of the final results. The results were defined as robust if the model remained significant in all single LOOA iterations at a *P* < 0.05. Significances are indicated by asterisks. For sensitivity analyses, surviving correlative outcome models were re-computed while iteratively taking various potentially confounding factors into account: (i) side of the lesion including its interaction with the regional volume to account for hemispheric specialization of basal ganglia^48-50^ and possible lateralization effects^51^ and differential plasticity rates^52^ known from traumatic brain injury patients; (ii) peak width of skeletonized mean diffusivity (PSMD) of the unaffected hemisphere as a marker of cerebral small vessel disease burden^53^; and (iii) influence of initial symptom severity onto volumetry–outcome relationship as operationalized by the interaction between NIHSS (dichotomized by median split into higher and lower NIHSS scores) and regional volume. For specificity analyses, we computed additional outcome models which included only global—and not regional—parameters of structural reserve that are (i) TIV; (ii) averaged grey subcortical matter volume; and (iii) averaged cortical grey matter volume of the unaffected hemisphere.

## Results

### Demographical and clinical data

**Figure 3:**
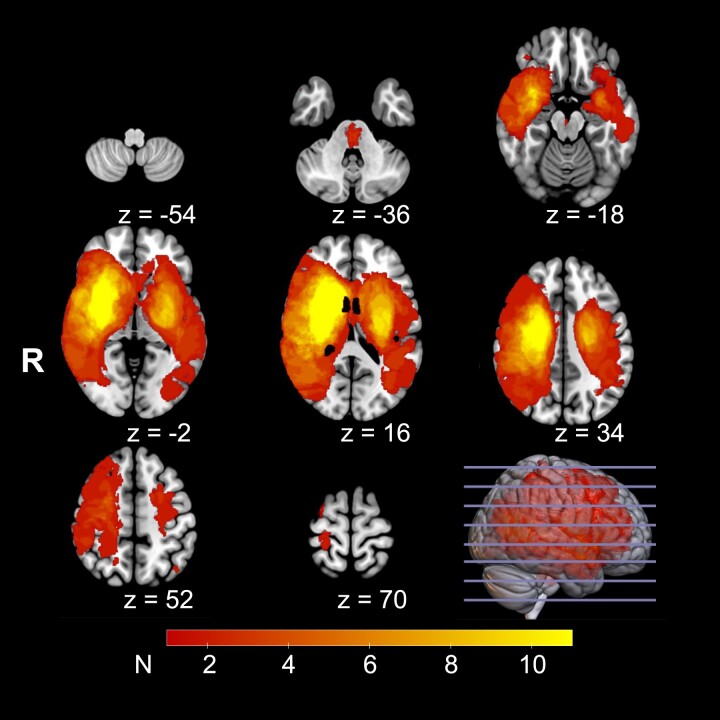
**Lesion distribution.** Heat map of stroke lesion distributions. Lesions overlay superimposed on a T1-weighted image in MNI standard space. *Z* values are shown for each axial slice. The colour represents the number (*N*) of patients affected per voxel. The letter R indicates the right side of the brain.

Data from 42 more severely impaired patients were analysed. The mean age of the stroke patients at examination was 70.8 years (standard deviation, SD = 12.4). Two (4.8%) patients were left-handed, 20 patients (47.6%) were female. The mean age in the control group was 70.3 years (SD = 10.1) and gender distribution was identical. There was no significant difference neither in age (*P* = 0.83), gender (*P* = 0.83) nor handedness (*P* = 0.79) between patients and controls. Twenty-six (61.9%) patients suffered from left-sided lesions. Mean lesion volume was 52.2 mL (SD = 68.8 mL, range: 0.6–303.3 mL). Median NIHSS at T1 was 8.5 (interquartile range, IQR 5.25–13.00), median mRS at T2 was 3 (IQR 1.25–4.00). Peak width of skeletonized mean diffusivity data for sensitivity analyses were available for 37 patients. Table 1 summarizes the demographic and clinical data. Individual patient data are given in Supplementary Table 1 and clinical characteristics by lesion side are shown in Supplementary Table 2. The topographic lesion distribution is shown in Fig. 3.

**Table 1:** Demographic and clinical data of the stroke patients

Parameter	Value
Age (mean, SD)	70.8, 12.4
Handedness (left, right)	2, 40
Gender (male, female)	22, 20
Lesion side (left, right)	16, 26
Lesion volume mL (mean, SD)	52.2, 68.8
NIHSS at T1 (median, IQR)	8.5, 5.25–13
mRS at T2 (median, IQR)	3, 1.25–4

Systematic overview of demographic and clinical data of the included patients including mean or median dependent on the parameter.

### Volumetry at baseline

Mean volumes of the seven regions of interest are shown in Table 2 along with the corresponding median for subsequent group separation into patients exhibiting larger and smaller volumes. Group comparison was conducted to assess any possible structural alterations present already at timepoint T1 which would complicate the interpretation of volumes as ‘pre-stroke’ brain reserve. There were no significant group differences in TIV, mesocorticolimbic structures, substantia nigra and caudate nucleus of the nigrostriatal pathway. Only the putamen showed a significant volume reduction at T1 in the stroke patients (mean 3.6 mL) when compared to controls (mean 4.1 mL, *P*_FDR_ = 0.032, Table 2).

**Table 2:** Group comparison of averaged volumes of interest

Pathway	Region/variable	Stroke mean [Median]	Control mean [Median]	*P*_FDR_
	Total intracranial volume	1519.216 [1517.300]	1479.242 [1454.415]	0.301
Mesocorticolimbic	Nucleus accumbens	0.740 [0.758]	0.773 [0.777]	0.262
	Medial PFC	11.946 [11.900]	12.483 [12.105]	0.262
	Amygdala	1.147 [1.202]	1.219 [1.232]	0.195
	Hippocampus	3.583 [3.633]	3.664 [3.572]	0.590
Nigrostriatal	Substantia nigra, pars compacta	0.013 [0.010]	0.013 [0.010]	0.701
	Caudate nucleus	2.969 [2.978]	3.201 [3.220]	0.195
	Putamen	3.619 [3.594]	4.058 [4.162]	0.032^a^

Mean [with median] baseline volumes (mL) of the mesolimbic structures of the unaffected hemisphere and the TIV are compared between stroke patients and controls. Notably, median values were used for group separation in patients exhibiting larger and smaller volumes. ^a^Indicates that the volume of the putamen was found to be significantly different between stroke patients and healthy controls.

### Volumetry–outcome relationship

**Figure 4:**
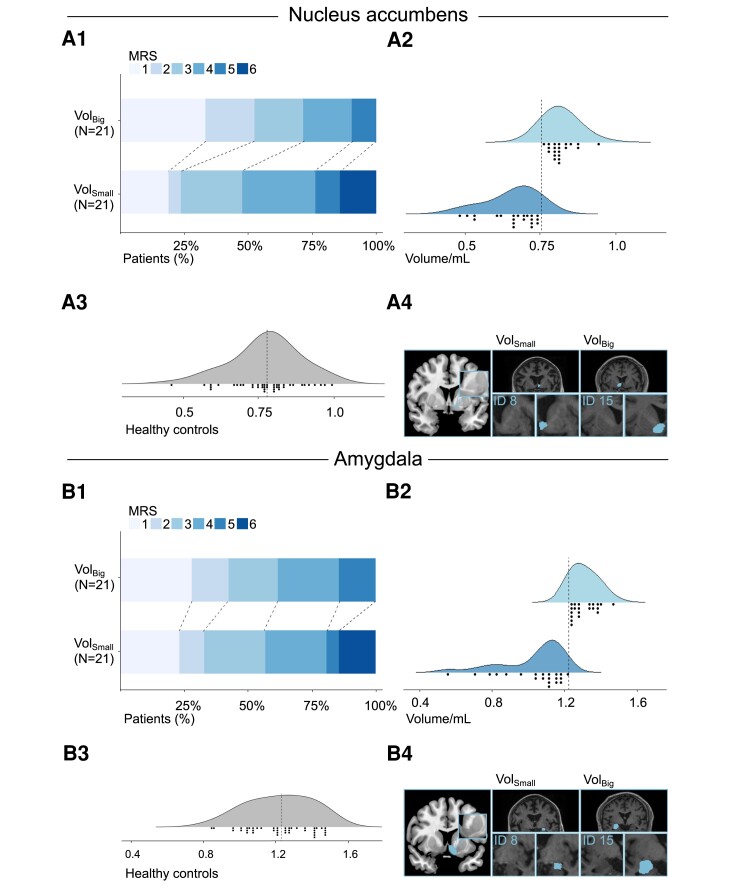
**Volumes of the nucleus accumbens and the amygdala and outcome after stroke.** Stacked histograms illustrate the distribution of mRS after 90 days for patients exhibiting bigger volumes (VOL_Big_, upper rows) and smaller volumes (VOL_Small_, lower rows) for the nucleus accumbens (**A1**) and the amygdala (**B1**). The mRS ranges from 0 to 6: 0: no symptoms; 1: no disability despite symptoms; 2: slight disability, unable to carry out all activities; 3: moderate disability, help required; 4: moderately severe disability, unable to walk; 5: severe disability, bedridden; 6: dead. Raincloud plots depict individual volumes for the nucleus accumbens (**A2**) and the amygdala (**B2**) for both volume groups. For comparison, volumes for healthy controls are given in **A3** and **B3**. Representative examples for big and small volumes for the nucleus accumbens (**A4**) and theamygdala (**B4**) are depicted by individual T1-weighted MRI images overlaid with the segmented regions of interest with the corresponding participant’s ID.

Ordinal logistic regression models showed significant associations between baseline grey matter volumes of the nucleus accumbens and the amygdala, and functional outcome after stroke. Specifically, patients exhibiting larger volumes in both regions showed a reduced odds of scoring higher on mRS at follow-up 3–6 months after stroke when compared to patients with smaller volumes (Nacc: OR = 0.176, *P*_FDR_ = 0.025, AMYG: OR = 0.183, *P*_FDR_ = 0.025, Table 3, Fig. 4). This association was independent of age, TIV, lesion volume and NIHSS at baseline. For additional sensitivity analyses, we re-computed the two winning models while considering several potentially confounding variables. First, possible hemispheric specialization of basal ganglia^48-50^ and possible lateralization effects^51^ and differential plasticity rates^52^ known from traumatic brain injury patients were addressed by including the interaction lesion side × regional volume. This interaction was not significant, neither for the nucleus accumbens (*P* = 0.78), not for the amygdala (*P* = 0.53) which argues against side effects regarding both the mesolimbic network assessed and the affected stroke hemisphere. Second, cerebral small vessel disease burden^53^ was assessed by incorporating PSMD data into the model. This did not alter the significant results for the nucleus accumbens and the amygdala. Third, the two winning models were extended by an interaction between NIHSS at baseline (binarized into higher and lower NIHSS scores) and the regional volume. This interaction was not significant (both *P* = 0.22) which argues against a dependence of the associations on initial symptom severity within the present cohort of severely impaired patients. The interplay between the volumes of the amygdala and the nucleus accumbens is illustrated in Supplementary Figure 1. To address region specificity of the present findings for the nucleus accumbens and the amygdala, we additionally explored the influence of global parameters of structural reserve to functional outcome, such as TIV, averaged grey subcortical matter volume and averaged cortical grey matter volume of the unaffected hemisphere. However, none of these measures showed a significant association (all *P* > 0.25).

**Table 3:** Structure–outcome relationship

Pathway	Region	OR (95% CI)	*P*_FDR_
Mesocorticolimbic	Nucleus accumbens	0.176 (0.043, 0.634)	0.025^a^
	Medial PFC	0.401 (0.120, 1.277)	0.142
	Amygdala	0.183 (0.049, 0.619)	0.025^a^
	Hippocampus	0.292 (0.080, 0.981)	0.108
Nigrostriatal	Substantia nigra, pars compacta	1.957 (0.608, 6.485)	0.260
	Caudate nucleus	0.397 (0.118, 1.268)	0.142
	Putamen	0.345 (0.100, 1.116)	0.133

Estimated ORs with 95% CIs for one level scoring higher in mRS at follow-up after 3–6 months after stroke in patients with larger volumes in mesocorticolimbic and nigrostriatal dopaminergic regions compared to patients with smaller regional volumes. Results are corrected for age, lesion volume, total intracranial volume and NIHSS at baseline. ^a^Indicates that larger volumes were significantly associated to favourable outcome.

## Discussion

The main finding of the present study was a significant positive association of larger volumes of the nucleus accumbens and the amygdala, key regions of the mesolimbic dopaminergic system, and a more favourable functional outcome 3–6 months after severe ischaemic stroke. This association was independent of the degree of initial impairment, age, lesion volume and TIV.

These findings add further evidence to the concept of structural reserve in the context of ischaemic stroke which proposes that larger reserve is associated with enhanced potential to recover.^11,14^ According to this concept, higher reserve equals increased neural substrate which in turn might influence both spontaneous neural repair, compensation and recovery mechanisms following stroke.^11,54^ Thus, the assessment of brain reserve may help to address and understand the differences in individual recovery from stroke and ease stroke outcome prediction.^13^

Structural reserve may relate to the global and focal level. On the global level, previous work has successfully linked the degree of white matter hyperintensities to worse functional outcome.^18,19^ On the focal level, other studies have shown that larger volumes of specific cerebellar lobules involved in motor and cognitive functioning, such as lobule IV, VI and VIIIB,^21^ or larger thicknesses of contralesional motor and non-motor cortices^22^ also carry valuable information regarding outcome variability after a severe stroke. The present results further add to these data by showing that structural reserve of distinct brain regions, the amygdala, and the nucleus accumbens, involved in dopaminergic neurotransmission across the motor and cognitive domain, particularly affecting motivational learning and reward processes, positively correlates with a more favourable outcome after stroke.

Our findings agree with previous animal data, studies in healthy participants and patient data. The nucleus accumbens is one of the key areas involved in motivation.^55^ In macaques, the recovery of dextrous finger movements after spinal cord injury at the cervical level was diminished by reversible pharmacological inactivation of the nucleus accumbens.^29^ In chronic stroke patients, smaller ipsilesional volumes of the nucleus accumbens correlated with worse sensorimotor functions.^56^ In acute stroke patients, lesions to this nucleus were associated with higher symptom burden and attenuated responses to specific training paradigms.^57^ Reward-related processes are another important domain influenced by dopaminergic neurotransmission via the mesocorticolimbic pathway, especially its mesolimbic part which includes the nucleus accumbens, the amygdala and the hippocampus.^38,58^ Reward correlates with better motor learning,^59,60^ procedural learning,^61^ and motor adaptation in healthy patients.^62^ In stroke patients, motor adaptation is also typically impaired. The addition of reward or punishment has been shown to improve motor adaptation in stroke patients.^63^ Finally, punishment has been reported to be strongly connected to fear memory formation, which depends on endogenous dopamine release in the amygdala.^64^ Collectively, these findings argue that reward and punishment-related processes could be influential for recovery after stroke. Our structural data extend these behavioural findings and show that surrogates of brain reserve in key areas along the underlying mesolimbic dopaminergic pathways might help to understand inter-subject variability in long-term outcome after stroke.

Mechanistically, there are two very interesting questions: First, would better reserve in dopaminergic key areas might enhance motivation, susceptibility to reward or punishment, and the compliance to rehabilitation on a behavioural, psychological level? Previous studies have shown that the degree of motivation in stroke patients promotes engagement and adherence, and it may increase the therapeutical effect of neurorehabilitation therapy.^25,26^ In patients with chronic traumatic brain injury, bilateral deep brain stimulation of the nucleus accumbens has shown to primarily impact on behavioural and emotional adjustment through prefrontal cortex modulation, which in turn improved functional independence even years after the injury.^65^ Second, would dopamine more directly act on cerebral reorganization and plastic processes? Ischaemic strokes alter the function of the dopaminergic system.^31^ For instance, dopamine release into the ipsilesional striatum shortly after stroke has been reported by some studies in rodents.^66,67^ The amount of released neurotransmitters has been found to correlate with stroke severity, outcome and age.^68^ Studies in gerbils and primates have suggested a negative link between higher releases of dopamine and a worse outcome.^68,69^ However, other authors have observed that spontaneous recovery in mice was linked to increased levels of dopamine in the contralesional hemisphere. Thus, they have argued that dopamine could be a promotor of recovery after stroke.^70^ This argumentation would be in line with animal data on motor cortex plasticity and motor skill learning^71^ or reports on traumatic brain injury which highlighted that dopaminergic neurotransmission is critically altered after the injury and dopamine levels and dopaminergic interventions have been linked with reductions in oxidative stress and improvement in cellular functions, neuronal survival and functional outcome.^72,73^ Given these considerations, the present data might indicate that enhanced dopamine reserve within the unaffected hemisphere may protect against stroke-induced damage and promote neuro-plastic processes on the cellular level.

So far, the translation of these promising preclinical data into the clinical application has been proven to be difficult. Dopaminergic interventions by drug therapy in clinical stroke cohorts have provided inconclusive results rendering a dopamine medication to improve recovery still a treatment approach which lacks clear evidence.^74-76^ Our present findings in more severely impaired stroke patients suggest that the response to dopaminergic interventions might depend on the level of impairment. Thus, future clinical trials might be warranted to include pre-specified subgroup analyses to explore the importance of impairment for treatment responses systematically.

Against our hypothesis, we did not find any significant associations between regional volumes along the mesocortical and nigrostriatal pathways and outcome. In rats, lesions to the medial prefrontal cortex have been reported to lead to selective but not generalized deficits in executive function.^77^ In humans, dopaminergic projections have been linked with higher cognitive functions such as top-down executive functions, self-knowledge, updating goal-directed behaviour and prediction of reward and motivation.^78-80^ On a speculative note, the outcome measure mRS as a global marker of disability could explain the absence of further associations. Future, prospective research focusing on more elaborated measures of activity of daily living or cognitive functioning are needed to investigate the importance of brain reserve of the dopaminergic systems for functional recovery after stroke in more detail. The same might hold true for the regions of the nigrostriatal pathway. Intuitively, this pathway appears even more relevant for motor functioning. It is involved in motor planning,^35^ habit formation^36^ and reward-driven motor learning.^28,55^ Direct lesions to the basal ganglia have been shown to associate with poor sensorimotor behaviour and recovery after stroke.^81,82^ One study in well-recovered stroke patients found that the amount of damage to nigrostriatal white matter tracts, quantified three months after stroke, correlated with worse fine motor skills after one year.^83^ Hence, a more detailed motor assessment including hand, finger and reach-to-grasp functions might be particularly needed to uncover structure–outcome associations for key areas along this pathway. A combined analysis of severely and mildly impaired patients would be needed to further address an impairment-specific importance of nigrostriatal pathways for stroke recovery.

There are several limitations worth noting. First, besides brain reserve, cognitive reserve is considered a potential predictor of individual stoke outcome, commonly operationalized by years of education.^14,17^ In this study, relevant cognitive impairment was an exclusion criterium. However, most patients, particularly of cohort C2, had very severe deficits early after stroke. A detailed cognitive assessment was neither included, nor would it have been feasible in this population. To what extent unknown pre-morbid cognitive dysfunction might have biased the present findings remains unclear and further distinction between brain reserve and cognitive reserve was limited in this work. Second, we have argued that inter-subject variability in brain reserve of motivational and reward networks could influence engagement and adherence to therapy throughout the rehabilitation period after stroke. Unfortunately, we do not have any information regarding the achieved amount of rehabilitation between T1 and T2. The inclusion of such additional information might alter the present modelling results. Future studies could extend our models by including detailed information, e.g. regarding the amount of inpatient rehabilitative training after discharge, the access to therapy in the outpatient or home-based setting, or even regarding the family background including daily support and motivation. Third, the primary lines of argumentation followed the concepts of dopaminergic motivational and reward processes. However, particularly the amygdala has been also linked, while exhibiting smaller volumes, to major depression^84^ and post-stroke depression.^85^ Unfortunately, to what extent features of depression might have influenced the present global recovery trajectories cannot be answered as detailed information was not acquired in the present cohorts. In turn, such structure–behaviour relationships might also indicate that early volume assessments in key areas of psychiatric diseases might enable personalized management targeting comorbidities, such as the occurrence of post-stroke depression and treatment regimes. Fourth, the small sample size could influence specificity and sensitivity negatively. To address this, we aimed at increasing the statistical power by dichotomization of the volume data in line with previous reports and fully corrected the results for multiple testing. Fifth, due to technical limitations, we could not include all areas of the dopaminergic system. The ventral tegmental area was excluded as it was not possible to extract the volumes properly. Sixth, the present analysis only focused on the unaffected hemisphere to exclude any direct lesion effects, in line with our previous analyses of contralesional cortices.^22^ Finally, 35 patients (83.33%) had lesions directly affecting ipsilesional dopaminergic regions, thus key regions of the mesolimbic pathways (Nacc: *N* = 12; mPFC: *N* = 13; AMYG: *N* = 24; HIP: *N* = 17; SNpc: *N* = 16; CAU: *N* = 34; PUT: *N* = 35). Consequently, in these patients, the ipsilesional mesolimbic pathway was directly affected. The influence of these direct lesion effects for the outcome inference remains unclear. This topic might be explored by future analyses systematically, potentially including tract-based markers of structural brain reserve of the dopaminergic system of the ipsilesional and contralesional hemisphere.

## Conclusion and future perspectives

The present study shows that larger volumes of distinct key areas of the dopaminergic network are positively associated with a better outcome after severe stroke. The data might suggest a link between the structural state of mesolimbic key areas contributing to motor learning, motivational and reward-related brain networks, and the success of neurorehabilitation, thereby promoting the emerging concepts of brain reserve capacity for stroke recovery to better understand outcome variability. Finally, the data could also provide novel evidence to reconsider dopaminergic interventions particularly in severely impaired stroke patients to enhance recovery after stroke given inconsistent effects in clinical trials until now.

## Supplementary Material

fcae122_Supplementary_Data

## Data Availability

Data will be made available by the authors upon reasonable request.

## References

[1] GBD 2019 Stroke Collaborators . Global, regional, and national burden of stroke and its risk factors, 1990–2019: A systematic analysis for the Global Burden of Disease Study 2019. Lancet Neurol. 2021;20(10):795–820. 34487721 10.1016/S1474-4422(21)00252-0 PMC8443449

[2] Grefkes C, Fink GR. Recovery from stroke: Current concepts and future perspectives. Neurol Res Pract. 2020;2:17. 33324923 10.1186/s42466-020-00060-6 PMC7650109

[3] Bonkhoff AK, Hope T, Bzdok D, et al Recovery after stroke: The severely impaired are a distinct group. J Neurol Neurosurg Psychiatry. 2022;93(4):369–378. 34937750 10.1136/jnnp-2021-327211

[4] Hayward KS, Schmidt J, Lohse KR, et al Are we armed with the right data? Pooled individual data review of biomarkers in people with severe upper limb impairment after stroke. Neuroimage Clin. 2017;13:310–319. 28053857 10.1016/j.nicl.2016.09.015 PMC5198729

[5] Koch P, Schulz R, Hummel FC. Structural connectivity analyses in motor recovery research after stroke. Ann Clin Transl Neurol. 2016;3(3):233–244. 27042683 10.1002/acn3.278 PMC4774263

[6] Rondina JM, Park CH, Ward NS. Brain regions important for recovery after severe post-stroke upper limb paresis. J Neurol Neurosurg Psychiatry. 2017;88(9):737–743. 28642286 10.1136/jnnp-2016-315030 PMC5561379

[7] Backhaus W, Braass H, Higgen FL, Gerloff C, Schulz R. Early parietofrontal network upregulation relates to future persistent deficits after severe stroke–a prospective cohort study. Brain Commun. 2021;3(2):fcab097. 34056601 10.1093/braincomms/fcab097 PMC8154858

[8] Bonkhoff AK, Espinoza FA, Gazula H, et al Acute ischaemic stroke alters the brain's preference for distinct dynamic connectivity states. Brain. 2020;143(5):1525–1540. 32357220 10.1093/brain/awaa101 PMC7241954

[9] Nemati PR, Backhaus W, Feldheim J, et al Brain network topology early after stroke relates to recovery. Brain Commun. 2022;4(2):fcac049. 35274100 10.1093/braincomms/fcac049 PMC8905614

[10] Hoonhorst MHJ, Nijland RHM, van den Berg PJS, Emmelot CH, Kollen BJ, Kwakkel G. Does transcranial magnetic stimulation have an added value to clinical assessment in predicting upper-limb function very early after severe stroke? Neurorehabil Neural Repair. 2018;32(8):682–690. 29972088 10.1177/1545968318785044 PMC6099969

[11] Rosenich E, Hordacre B, Paquet C, Koblar SA, Hillier SL. Cognitive reserve as an emerging concept in stroke recovery. Neurorehabil Neural Repair. 2020;34(3):187–199. 32089097 10.1177/1545968320907071

[12] Stern Y, Barulli D. Cognitive reserve. Handb Clin Neurol. 2019;167:181–190. 31753132 10.1016/B978-0-12-804766-8.00011-X

[13] Umarova RM, Gallucci L, Hakim A, Wiest R, Fischer U, Arnold M. Adaptation of the concept of brain reserve for the prediction of stroke outcome: Proxies, neural mechanisms, and significance for research. Brain Sci. 2024;14:77. 38248292 10.3390/brainsci14010077 PMC10813468

[14] Satz P . Brain reserve capacity on symptom onset after brain injury: A formulation and review of evidence for threshold theory. Neuropsychology. 1993;7(3):273–295.

[15] Bretzner M, Bonkhoff AK, Schirmer MD, et al Radiomics-derived brain age predicts functional outcome after acute ischemic stroke. Neurology. 2023;100(8):e822–e833. 36443016 10.1212/WNL.0000000000201596 PMC9984219

[16] Bu N, Khlif MS, Lemmens R, et al Imaging markers of brain frailty and outcome in patients with acute ischemic stroke. Stroke. 2021;52(3):1004–1011. 33504185 10.1161/STROKEAHA.120.029841

[17] Umarova RM, Schumacher LV, Schmidt CSM, et al Interaction between cognitive reserve and age moderates effect of lesion load on stroke outcome. Sci Rep. 2021;11(1):4478. 33627742 10.1038/s41598-021-83927-1 PMC7904829

[18] Etherton MR, Schirmer MD, Zotin MCZ, et al Global white matter structural integrity mediates the effect of age on ischemic stroke outcomes. Int J Stroke. 2021;2021 Nov 3:17474930211055906. 10.1177/17474930211055906 34730044

[19] Hong S, Giese AK, Schirmer MD, et al Excessive white matter hyperintensity increases susceptibility to poor functional outcomes after acute ischemic stroke. Front Neurol. 2021;12:700616. 34566844 10.3389/fneur.2021.700616 PMC8461233

[20] Schirmer MD, Donahue KL, Nardin MJ, et al Brain volume: An important determinant of functional outcome after acute ischemic stroke. Mayo Clin Proc. 2020;95(5):955–965. 32370856 10.1016/j.mayocp.2020.01.027

[21] Sadeghihassanabadi F, Frey BM, Backhaus W, et al Structural cerebellar reserve positively influences outcome after severe stroke. Brain Commun. 2022;4(6):fcac203. 36337341 10.1093/braincomms/fcac203 PMC9629400

[22] Rojas Albert A, Backhaus W, Graterol Perez JA, et al Cortical thickness of contralesional cortices positively relates to future outcome after severe stroke. Cereb Cortex. 2022;32(24):5622–5627. 35169830 10.1093/cercor/bhac040

[23] Krakauer JW . Motor learning: Its relevance to stroke recovery and neurorehabilitation. Curr Opin Neurol. 2006;19(1):84–90. 16415682 10.1097/01.wco.0000200544.29915.cc

[24] Dipietro L, Krebs HI, Volpe BT, et al Learning, not adaptation, characterizes stroke motor recovery: Evidence from kinematic changes induced by robot-assisted therapy in trained and untrained task in the same workspace. IEEE Trans Neural Syst Rehabil Eng. 2012;20(1):48–57. 22186963 10.1109/TNSRE.2011.2175008 PMC4687974

[25] Wulf G, Lewthwaite R. Optimizing performance through intrinsic motivation and attention for learning: The OPTIMAL theory of motor learning. Psychon Bull Rev. 2016;23(5):1382–1414. 26833314 10.3758/s13423-015-0999-9

[26] Gangwani R, Cain A, Collins A, Cassidy JM. Leveraging factors of self-efficacy and motivation to optimize stroke recovery. Front Neurol. 2022;13:823202. 35280288 10.3389/fneur.2022.823202 PMC8907401

[27] Widmer M, Held JPO, Wittmann F, et al Reward during arm training improves impairment and activity after stroke: A randomized controlled trial. Neurorehabil Neural Repair. 2022;36(2):140–150. 34937456 10.1177/15459683211062898 PMC8796156

[28] Vassiliadis P, Derosiere G, Dubuc C, et al Reward boosts reinforcement-based motor learning. iScience. 2021;24(7):102821. 34345810 10.1016/j.isci.2021.102821 PMC8319366

[29] Sawada M, Kato K, Kunieda T, et al Function of the nucleus accumbens in motor control during recovery after spinal cord injury. Science. 2015;350(6256):98–101. 26430122 10.1126/science.aab3825

[30] Oldehinkel M, Llera A, Faber M, et al Mapping dopaminergic projections in the human brain with resting–state fMRI. Elife. 2022:11:e71846. 10.7554/eLife.71846 PMC8843090 35113016

[31] Gower A, Tiberi M. The intersection of central dopamine system and stroke: Potential avenues aiming at enhancement of motor recovery. Front Synaptic Neurosci. 2018;10:18. 30034335 10.3389/fnsyn.2018.00018 PMC6043669

[32] Bromberg-Martin ES, Matsumoto M, Hikosaka O. Dopamine in motivational control: Rewarding, aversive, and alerting. Neuron. 2010;68(5):815–834. 21144997 10.1016/j.neuron.2010.11.022 PMC3032992

[33] Bjorklund A, Dunnett SB. Dopamine neuron systems in the brain: An update. Trends Neurosci. 2007;30(5):194–202. 17408759 10.1016/j.tins.2007.03.006

[34] Masato A, Plotegher N, Boassa D, Bubacco L. Impaired dopamine metabolism in Parkinson’s disease pathogenesis. Mol Neurodegener. 2019;14(1):35. 31488222 10.1186/s13024-019-0332-6 PMC6728988

[35] Joshua M, Adler A, Bergman H. The dynamics of dopamine in control of motor behavior. Curr Opin Neurobiol. 2009;19(6):615–620. 19896833 10.1016/j.conb.2009.10.001

[36] Faure A, Haberland U, Conde F, El Massioui N. Lesion to the nigrostriatal dopamine system disrupts stimulus-response habit formation. J Neurosci. 2005;25(11):2771–2780. 15772337 10.1523/JNEUROSCI.3894-04.2005 PMC6725127

[37] Arias-Carrion O, Stamelou M, Murillo-Rodriguez E, Menendez-Gonzalez M, Poppel E. Dopaminergic reward system: A short integrative review. Int Arch Med. 2010;3:24. 20925949 10.1186/1755-7682-3-24 PMC2958859

[38] Ruiz-Tejada A, Neisewander J, Katsanos CS. Regulation of voluntary physical activity behavior: A review of evidence involving dopaminergic pathways in the brain. Brain Sci. 2022;12(3):333. 35326289 10.3390/brainsci12030333 PMC8946175

[39] Quessy F, Bittar T, Blanchette LJ, Levesque M, Labonte B. Stress-induced alterations of mesocortical and mesolimbic dopaminergic pathways. Sci Rep. 2021;11(1):11000. 34040100 10.1038/s41598-021-90521-y PMC8154906

[40] Khastkhodaei Z, Muthuraman M, Yang JW, Groppa S, Luhmann HJ. Functional and directed connectivity of the cortico-limbic network in mice in vivo. Brain Struct Funct. 2021;226(3):685–700. 33442810 10.1007/s00429-020-02202-7 PMC7981333

[41] Gudelsky GA . Tuberoinfundibular dopamine neurons and the regulation of prolactin secretion. Psychoneuroendocrinology. 1981;6(1):3–16. 7017786 10.1016/0306-4530(81)90044-5

[42] Bonstrup M, Krawinkel L, Schulz R, et al Low-frequency brain oscillations track motor recovery in human stroke. Ann Neurol. 2019;86(6):853–865. 31604371 10.1002/ana.25615

[43] Bernhardt J, Hayward KS, Kwakkel G, et al Agreed definitions and a shared vision for new standards in stroke recovery research: The stroke recovery and rehabilitation roundtable taskforce. Neurorehabil Neural Repair. 2017;31(9):793–799. 28934920 10.1177/1545968317732668

[44] Gaser C. Manual computational anatomy toolbox-CAT12. *Struct Brain Mapping Group at the Departments of Psychiatry and Neurology*, University of Jena. Accessed 12 April 2023. http://www.neuro.uni-jena.de/cat/index.html

[45] Ashburner J . A fast diffeomorphic image registration algorithm. Neuroimage. 2007;38(1):95–113. 17761438 10.1016/j.neuroimage.2007.07.007

[46] Rolls ET, Huang CC, Lin CP, Feng J, Joliot M. Automated anatomical labelling atlas 3. Neuroimage. 2020;206:116189. 31521825 10.1016/j.neuroimage.2019.116189

[47] Venables WN, Ripley BD. Modern applied statistics with S. Springer; 2002.

[48] Lubben N, Ensink E, Coetzee GA, Labrie V. The enigma and implications of brain hemispheric asymmetry in neurodegenerative diseases. Brain Commun. 2021;3(3):fcab211. 34557668 10.1093/braincomms/fcab211 PMC8454206

[49] Glick SD, Ross DA, Hough LB. Lateral asymmetry of neurotransmitters in human brain. Brain Res. 1982;234(1):53–63. 6120746 10.1016/0006-8993(82)90472-3

[50] Kooistra CA, Heilman KM. Motor dominance and lateral asymmetry of the globus pallidus. Neurology. 1988;38(3):388–390. 3347342 10.1212/wnl.38.3.388

[51] Aamodt EB, Lydersen S, Alnaes D, et al Longitudinal brain changes after stroke and the association with cognitive decline. Front Neurol. 2022;13:856919. 35720079 10.3389/fneur.2022.856919 PMC9204010

[52] Dennis EL, Newsome MR, Lindsey HM, et al Altered lateralization of the cingulum in deployment-related traumatic brain injury: An ENIGMA military-relevant brain injury study. Hum Brain Mapp. 2023;44(5):1888–1900. 36583562 10.1002/hbm.26179 PMC9980891

[53] Baykara E, Gesierich B, Adam R, et al A novel imaging marker for small vessel disease based on skeletonization of white matter tracts and diffusion histograms. Ann Neurol. 2016;80(4):581–592. 27518166 10.1002/ana.24758

[54] Langhorne P, Bernhardt J, Kwakkel G. Stroke rehabilitation. Lancet. 2011;377(9778):1693–1702. 21571152 10.1016/S0140-6736(11)60325-5

[55] Widmer M, Lutz K, Luft AR. Reduced striatal activation in response to rewarding motor performance feedback after stroke. Neuroimage Clin. 2019;24:102036. 31698315 10.1016/j.nicl.2019.102036 PMC6978223

[56] Liew SL, Zavaliangos-Petropulu A, Schweighofer N, et al Smaller spared subcortical nuclei are associated with worse post-stroke sensorimotor outcomes in 28 cohorts worldwide. Brain Commun. 2021;3(4):fcab254. 34805997 10.1093/braincomms/fcab254 PMC8598999

[57] Skidmore ER, Shih M, Terhorst L, O’Connor EE. Lesion location may attenuate response to strategy training in acute stroke. PM R. 2022;14(3):329–336. 33728742 10.1002/pmrj.12590 PMC8446102

[58] Lammel S, Lim BK, Malenka RC. Reward and aversion in a heterogeneous midbrain dopamine system. Neuropharmacology. 2014;76 Pt B(0 0):351–359. 23578393 10.1016/j.neuropharm.2013.03.019 PMC3778102

[59] Abe M, Schambra H, Wassermann EM, Luckenbaugh D, Schweighofer N, Cohen LG. Reward improves long-term retention of a motor memory through induction of offline memory gains. Curr Biol. 2011;21(7):557–562. 21419628 10.1016/j.cub.2011.02.030 PMC3075334

[60] Widmer M, Ziegler N, Held J, Luft A, Lutz K. Rewarding feedback promotes motor skill consolidation via striatal activity. Prog Brain Res. 2016;229:303–323. 27926445 10.1016/bs.pbr.2016.05.006

[61] Wachter T, Lungu OV, Liu T, Willingham DT, Ashe J. Differential effect of reward and punishment on procedural learning. J Neurosci. 2009;29(2):436–443. 19144843 10.1523/JNEUROSCI.4132-08.2009 PMC2765863

[62] Galea JM, Mallia E, Rothwell J, Diedrichsen J. The dissociable effects of punishment and reward on motor learning. Nat Neurosci. 2015;18(4):597–602. 25706473 10.1038/nn.3956

[63] Quattrocchi G, Greenwood R, Rothwell JC, Galea JM, Bestmann S. Reward and punishment enhance motor adaptation in stroke. J Neurol Neurosurg Psychiatry. 2017;88(9):730–736. 28377451 10.1136/jnnp-2016-314728

[64] Frick A, Bjorkstrand J, Lubberink M, Eriksson A, Fredrikson M, Ahs F. Dopamine and fear memory formation in the human amygdala. Mol Psychiatry. 2022;27(3):1704–1711. 34862441 10.1038/s41380-021-01400-x PMC9095491

[65] Rezai AR, Sederberg PB, Bogner J, et al Improved function after deep brain stimulation for chronic, severe traumatic brain injury. Neurosurgery. 2016;79(2):204–211. 26702839 10.1227/NEU.0000000000001190

[66] Globus MY, Busto R, Dietrich WD, Martinez E, Valdes I, Ginsberg MD. Effect of ischemia on the in vivo release of striatal dopamine, glutamate, and gamma-aminobutyric acid studied by intracerebral microdialysis. J Neurochem. 1988;51(5):1455–1464. 2902196 10.1111/j.1471-4159.1988.tb01111.x

[67] Hashimoto N, Matsumoto T, Mabe H, Hashitani T, Nishino H. Dopamine has inhibitory and accelerating effects on ischemia-induced neuronal cell damage in the rat striatum. Brain Res Bull. 1994;33(3):281–288. 7904889 10.1016/0361-9230(94)90195-3

[68] Delbarre B, Delbarre G, Calinon F. Free radicals and neurotransmitters in gerbil brain. Influence of age and ischemia reperfusion insult. EXS. 1992;62:199–212. 1360281 10.1007/978-3-0348-7460-1_20

[69] Richards DA, Obrenovitch TP, Symon L, Curzon G. Extracellular dopamine and serotonin in the rat striatum during transient ischaemia of different severities: A microdialysis study. J Neurochem. 1993;60(1):128–136. 8417136 10.1111/j.1471-4159.1993.tb05830.x

[70] Obi K, Amano I, Takatsuru Y. Role of dopamine on functional recovery in the contralateral hemisphere after focal stroke in the somatosensory cortex. Brain Res. 2018;1678:146–152. 29079503 10.1016/j.brainres.2017.10.022

[71] Rioult-Pedotti MS, Pekanovic A, Atiemo CO, Marshall J, Luft AR. Dopamine promotes motor cortex plasticity and motor skill learning via PLC activation. PLoS One. 2015;10(5):e0124986. 25938462 10.1371/journal.pone.0124986 PMC4418826

[72] Jenkins PO, De Simoni S, Bourke NJ, et al Dopaminergic abnormalities following traumatic brain injury. Brain. 2018;141(3):797–810. 29360949 10.1093/brain/awx357

[73] Chen YH, Huang EY, Kuo TT, Miller J, Chiang YH, Hoffer BJ. Impact of traumatic brain injury on dopaminergic transmission. Cell Transplant. 2017;26(7):1156–1168. 28933212 10.1177/0963689717714105 PMC5657731

[74] Ford GA, Bhakta BB, Cozens A, et al Safety and efficacy of co-careldopa as an add-on therapy to occupational and physical therapy in patients after stroke (DARS): A randomised, double-blind, placebo-controlled trial. Lancet Neurol. 2019;18(6):530–538. 31122493 10.1016/S1474-4422(19)30147-4 PMC6527868

[75] Scheidtmann K, Fries W, Muller F, Koenig E. Effect of levodopa in combination with physiotherapy on functional motor recovery after stroke: A prospective, randomised, double–blind study. Lancet. 2001;358(9284):787–790. 11564483 10.1016/S0140-6736(01)05966-9

[76] Stinear CM . Dopamine for motor recovery after stroke: Where to from here? Lancet Neurol. 2019;18(6):514–515. 31122485 10.1016/S1474-4422(19)30162-0

[77] Deziel RA, Ryan CL, Tasker RA. Ischemic lesions localized to the medial prefrontal cortex produce selective deficits in measures of executive function in rats. Behav Brain Res. 2015;293:54–61. 26166190 10.1016/j.bbr.2015.07.003

[78] Jobson DD, Hase Y, Clarkson AN, Kalaria RN. The role of the medial prefrontal cortex in cognition, ageing and dementia. Brain Commun. 2021;3(3):fcab125. 34222873 10.1093/braincomms/fcab125 PMC8249104

[79] Jones DT, Graff-Radford J. Executive dysfunction and the prefrontal cortex. Continuum (Minneap Minn). 2021;27(6):1586–1601. 34881727 10.1212/CON.0000000000001009

[80] Myers CA, Wang C, Black JM, Bugescu N, Hoeft F. The matter of motivation: Striatal resting-state connectivity is dissociable between grit and growth mindset. Soc Cogn Affect Neurosci. 2016;11(10):1521–1527. 27217105 10.1093/scan/nsw065 PMC5040906

[81] Boyd LA, Winstein CJ. Providing explicit information disrupts implicit motor learning after basal ganglia stroke. Learn Mem. 2004;11(4):388–396. 15286181 10.1101/lm.80104 PMC498316

[82] Fries W, Danek A, Scheidtmann K, Hamburger C. Motor recovery following capsular stroke. Role of descending pathways from multiple motor areas. Brain. 1993;116(Pt 2):369–382. 8461971 10.1093/brain/116.2.369

[83] Rimmele DL, Frey BM, Cheng B, et al Association of extrapyramidal tracts’ integrity with performance in fine motor skills after stroke. Stroke. 2018;49(12):2928–2932. 30571408 10.1161/STROKEAHA.118.022706

[84] Nolan M, Roman E, Nasa A, et al Hippocampal and amygdalar volume changes in major depressive disorder: A targeted review and focus on stress. Chronic Stress (Thousand Oaks). 2020;4:2470547020944553. 10.1177/2470547020944553 PMC7513405 33015518

[85] Loubinoux I, Kronenberg G, Endres M, et al Post-stroke depression: Mechanisms, translation and therapy. J Cell Mol Med. 2012;16(9):1961–1969. 22348642 10.1111/j.1582-4934.2012.01555.x PMC3822966

