# Correction to: Dopaminergic mesolimbic structural reserve is positively linked to better outcome after severe stroke

**DOI:** 10.1093/braincomms/fcae191

**Published:** 2024-06-06

This is a correction to: Liv Asmussen, Benedikt M Frey, Lukas K Frontzkowski, Paweł P Wróbel, L Sophie Grigutsch, Chi-un Choe, Marlene Bönstrup, Bastian Cheng, Götz Thomalla, Fanny Quandt, Christian Gerloff, Robert Schulz, Dopaminergic mesolimbic structural reserve is positively linked to better outcome after severe stroke, *Brain Communications*, Volume 6, Issue 3, 2024, fcae122, https://doi.org/10.1093/braincomms/fcae122

In Figure 4, the ordering of plots for the subgroups of patients exhibiting big and small volumes for nucleus accumbens (A2) and amygdala (B2) has been corrected to match the layout of panels A1 and B1 (e.g., patients with smaller volumes in the lower position).

