# Supplementary material for: Dopaminergic mesolimbic structural reserve is positively linked to better outcome after severe stroke
Source: Brain Commun. 2024 Apr 9;6(3):fcae122. doi: 10.1093/braincomms/fcae122 (PMC11073754; doi:10.1093/braincomms/fcae122)
Supplement: fcae122_Supplementary_Data [file fcae122_supplementary_data.docx]

# Supplementary material

**Supplementary Table 1 Clinical and demographic data of the patients**

| **ID** | **Cohort** | **Age** | **Sex** | **Handedness** | **Lesion side** | **Lesion**  **volume (mL)** | **Post. Circ.** | **Thrombolysis/**  **Thrombectomy (mTICI)** | **NIHSS T1** | **mRS T2** | **T2** |
| --- | --- | --- | --- | --- | --- | --- | --- | --- | --- | --- | --- |
| 1 | C1 | 49 | female | right | left | 53.80 | no | Yes/Yes (-) | 10 | 2 | 3 |
| 2 | C1 | 73 | female | right | left | 5.80 | no | No/No | 9 | 4 | 3 |
| 3 | C1 | 65 | male | left | left | 6.60 | no | No/No | 8 | 3 | 3 |
| 4 | C1 | 81 | male | right | left | 0.60 | yes | Yes/No | 4 | 1 | 3 |
| 5 | C1 | 81 | male | right | left | 1.70 | no | No/No | 4 | 3 | 3 |
| 6 | C1 | 76 | male | right | left | 1.70 | no | No/No | 5 | 1 | 3 |
| 7 | C1 | 48 | male | right | left | 24.40 | no | Yes/Yes (3) | 7 | 2 | 3 |
| 8 | C1 | 87 | female | right | left | 1.00 | no | No/No | 1 | 1 | 3 |
| 9 | C1 | 83 | female | right | left | 3.30 | no | No/No | 5 | 4 | 3 |
| 10 | C1 | 43 | male | right | right | 79.80 | no | Yes/Yes (3) | 13 | 2 | 3 |
| 11 | C1 | 56 | male | right | right | 2.50 | no | No/No | 13 | 4 | 3 |
| 12 | C1 | 69 | male | left | right | 25.10 | no | Yes/Yes (3) | 3 | 1 | 3 |
| 13 | C1 | 73 | female | right | right | 26.80 | no | Yes/Yes (-) | 3 | 1 | 3 |
| 14 | C1 | 86 | female | right | right | 10.70 | no | No/No | 5 | 2 | 3 |
| 15 | C1 | 58 | male | right | right | 0.70 | no | No/No | 7 | 1 | 3 |
| 16 | C1 | 50 | male | right | right | 25.50 | no | Yes/Yes (2b) | 7 | 2 | 3 |
| 17 | C1 | 77 | female | right | right | 9.10 | no | No/No | 8 | 3 | 3 |
| 18 | C1 | 70 | female | right | right | 74.40 | no | Yes/Yes (2a) | 5 | 1 | 3 |
| 19 | C1 | 85 | female | right | right | 16.70 | no | No/No | 7 | 4 | 3 |
| 20 | C1 | 47 | male | right | right | 2.60 | no | Yes/No | 6 | 3 | 3 |
| 21 | C1 | 59 | male | right | right | 14.30 | no | No/No | 7 | 4 | 3 |
| 22 | C1 | 50 | male | right | right | 50.10 | no | Yes/Yes (3) | 4 | 1 | 3 |
| 23 | C2 | 78 | male | right | left | 58.10 | no | No/No | 17 | 5 | 3 |
| 24 | C2 | 83 | female | right | left | 101.40 | yes | No/No | 20 | 6 | 3 |
| 25 | C2 | 63 | male | right | left | 55.80 | no | No/No | 13 | 1 | 3 |
| 26 | C2 | 73 | female | right | left | 14.40 | no | Yes/Yes (2b) | 9 | 3 | 6 |
| 27 | C2 | 80 | female | right | left | 20.50 | no | No/Yes (-) | 11 | 4 | 6 |
| 28 | C2 | 78 | female | right | left | 33.60 | no | No/No | 10 | 3 | 3 |
| 29 | C2 | 74 | male | right | left | 303.30 | no | Yes/Yes (2b) | 24 | 5 | 3 |
| 30 | C2 | 76 | male | right | right | 101.00 | no | Yes/No (3) | 11 | 3 | 3 |
| 31 | C2 | 71 | male | right | right | 75.20 | no | No/Yes (-) | 15 | 6 | 6 |
| 32 | C2 | 77 | female | right | right | 286.70 | no | Yes/No | 11 | 4 | 6 |
| 33 | C2 | 71 | female | right | right | 38.40 | no | Yes/Yes (2a) | 9 | 3 | 3 |
| 34 | C2 | 58 | male | right | right | 98.00 | yes | Yes/Yes (-) | 13 | 5 | 3 |
| 35 | C2 | 67 | female | right | right | 7.40 | no | Yes/No | 11 | 1 | 6 |
| 36 | C2 | 80 | male | right | right | 108.40 | no | Yes/Yes (0) | 16 | 6 | 3 |
| 37 | C2 | 79 | female | right | right | 120.40 | no | Yes/Yes (2b) | 8 | 4 | 3 |
| 38 | C2 | 85 | female | right | right | 33.50 | no | No/Yes (2b) | 15 | 5 | 3 |
| 39 | C2 | 78 | male | right | right | 178.10 | no | Yes/Yes (3) | 17 | 4 | 3 |
| 40 | C2 | 73 | female | right | right | 27.60 | no | Yes/Yes (2a) | 5 | 1 | 3 |
| 41 | C2 | 76 | male | right | right | 91.80 | yes | Yes/Yes (-) | 15 | 4 | 6 |
| 42 | C2 | 89 | female | right | right | 2.60 | yes | Yes/No | 7 | 3 | 3 |

**Post. Circ.** Stroke location in posterior circulation including brain stem. **mTICI** thrombolysis in cerebral infarction grading system, partial perfusion of the treated vessel is reached in grade 2B, - indicates missing value for mTICI. **T1** Baseline NIHSS value was obtained in the acute stage after stroke (3-14 days). **T2** Follow-up clinical data collection in the late sub-acute stage 3-6 months after stroke as indicated. Except for ID 3 and 12, all patients were right-handed.

**Supplementary Table 2 Clinical characteristics by lesion side**

| **Lesionside** | **All** | **Left (N=16)** | **Right (N= 26)** | ***P*** |
| --- | --- | --- | --- | --- |
| Age (mean, SD) | 70.8, 12.4 | 73.25, 11.5 | 69.35, 12.9 | 0.32 |
| Gender (male, female) | 22, 20 | 8, 8 | 14, 12 | 1 |
| Handedness (left, right) | 2, 40 | 1, 15 | 1, 25 | 1 |
| Lesionvolume (mean, SD) | 52.2, 68.8 | 42.9, 75.2 | 58, 65.5 | 0.51 |
| NIHSS T1 (median, IQR) | 8.5, 5.25-13 | 9.0, 5-11.5 | 8.0, 6.25-13 | 0.76 |
| mRS T2 (median, IQR) | 3, 1.25 - 4 | 3, 1.75-4 | 3, 1.25-4 | 0.98 |

T-tests were conducted to identify any significant differences in age, NIHSS T1, and stroke lesion volume.

Fisher’s exact tests were used for the other variables.

**
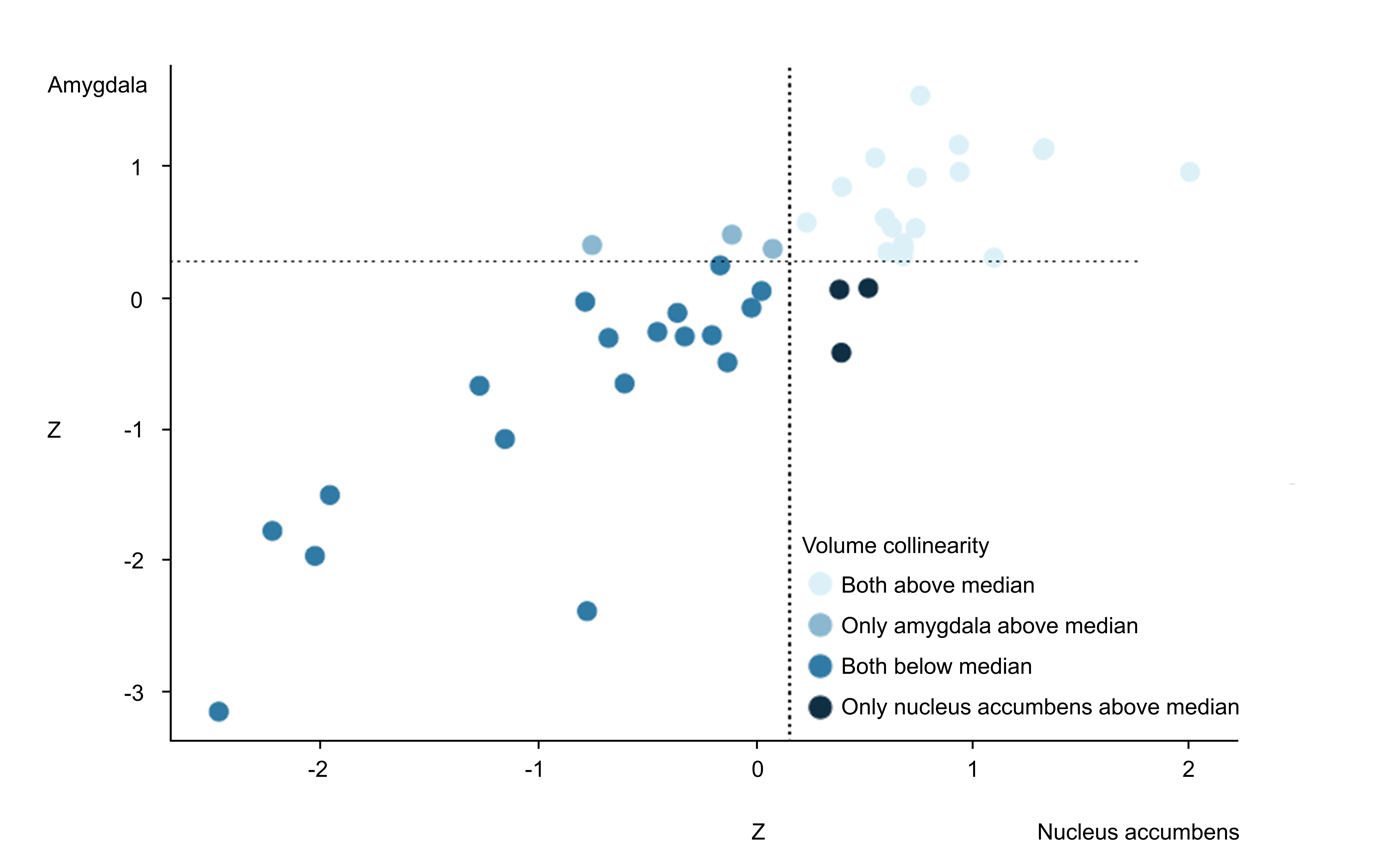
**

**Supplementary Figure 1 Scatter plot illustrating the interplay between amygdala and nucleus accumbens volumes**

The scatter plot shows the interplay between amygdala and nucleus accumbens volumes converted into Z-scores. The dotted lines mark the median. Each data point shows the individual volumes of Amygdala and Nucleus accumbens and indicates whether this volume is above or below median.
